# Simulating surveys for graduate researcher development

**DOI:** 10.3389/frma.2024.1360333

**Published:** 2024-05-01

**Authors:** Phillip Allen Morris, Elisa Thompson

**Affiliations:** Educational Leadership, Research, and Policy, University of Colorado Colorado Springs, Colorado Springs, CO, United States

**Keywords:** survey design, graduate student research, statistics education, survey methods, item development, survey simulation, data simulation

## Abstract

Recognizing the value of experiential education in social/behavioral science research training, we designed and offered a simulation of a survey research project for doctoral students in education. Through three phases of the project, from instrument design through scale investigation and quantitative analyses, students are developed as researchers in a realistic and authentic way. In this paper, we highlight the advantages, challenges, and outcomes from applying simulation methods within graduate research methods courses, with a specific focus on survey methodology and quantitative skill development.

## Introduction

Due to the many applications and frequent use of surveys for collecting data, the facility to design psychometrically sound surveys is a critical skill required of graduate students in the social sciences. Survey research is critical to many types of research, from community-based participatory research involving youth in developing a survey on the local availability of sexual health resources in their community (Flicker et al., 2010) to a cross-cultural, large-scale assessment of the role of instructional quality and classroom relationships with students on teacher satisfaction (Harrison et al., 2023). As ubiquitous as their use, are warnings about the difficulty of creating strong surveys and guidance about how to avoid survey design pitfalls (Singh et al., 2009; Robb and Shellenbarger, 2020).

Dillman et al. (2014), contend that designing surveys that reduce the burden and increase the benefit to respondents is a complex process involving the intentional application of social exchange theory and actions encouraging participants to complete the survey. Intentional practices, such as reducing length and complexity of the survey while enticing the respondent with features such as multiple modes of responding, association with a reputable, well-known sponsor, and minimizing the collection of sensitive or personal information, are all critical components in producing an effective instrument (Dillman et al., 2014). According to Shultz et al. (2021) survey construction includes myriad tasks such as deciding upon open or closed-ended items, deciding about item characteristics such as ordering of items and including or omitting a “Don't know” response, piloting the survey, and administration practices that maximize response rates. These skills are difficult to master without hands-on experience.

To this end, the Guidelines for Assessment and Instruction in Statistics Education (GAISE, 2016) recommends instructors integrate real data with a context and purpose, foster active learning, and use technology to analyze data. Using data that provides a context and purpose to the student is engaging (Oliver et al., 2018) and allows students to gain experience with the complexities of “real” data (GAISE, 2016). Master's level principal preparation students indicated favoring methods like these in a survey of preferred instructional methods because they preferred problem-based learning where assignments were complex and directly related to future professional endeavors (Oliver et al., 2018). Further, the preference for experiential learning assignments that include technology may be especially strong among learners from the Millennial and Gen Z age groups (Ortega-Dela Cruz, 2020).

Other researchers have demonstrated the value of using simulated data as an effective means for teaching statistical concepts and analyses. Fellers and Kuiper (2020) contend that students are encountering more complex data analyses in professional publications and the popular media, but they not only fail to fully understand the implications of the results, they also cannot vet the sources for psychometric fidelity. The authors assert that using simulated data provides students an opportunity to obtain hands on experience with complex data. Witte (2017) found that allowing students to learn statistical analysis through hands-on manipulation of “messy” data is more effective than approaches relying on theory, computations, and data that is cleaned and prepped to facilitate easy statistical analysis. Results indicating significantly better learning outcomes using data simulations compared to traditional teaching methods have also been demonstrated in finance investment university classes (Chulkov and Wang, 2020). The benefit of experiencing real world statistical analysis complexities was also reported by Baglin et al. (2013) and Birrell (2020) who found students were more engaged when using the data simulation program*, Island*, which allowed them to experience simulated participants exhibiting complex behaviors like refusing to give consent or providing inconsistent responses on retest items. Recognizing the value of experiential education in social/behavioral science research training, the authors developed and applied a survey simulation project for PhD students enrolled in an Education Leadership and Policy program. The survey design simulation, described herein, provides high levels of context and purpose while also fostering skills necessary for successfully completing an independent research project. In addition to foundational design and analysis considerations, aspects of the assignment require the use of statistical software allowing them to develop facility with technology tools used in the field of educational research and leadership (GAISE, 2016). The purpose of this paper is to provide a guide for simulating a complete survey research project, and underscores the advantages, challenges, and outcomes from applying simulation methods within graduate level curriculum.

## Survey design for emerging researchers

The survey simulation project is designed to introduce students to survey research from initial research conception to final presentation/publication, including (1) designing an instrument, (2) presentation of a deployment plan, sampling considerations, and power analyses, (3) creation of a codebook, (4) reliability/scale analyses, (5) exploratory factor analyses, (6) research question creation, (7) data analysis to answer research questions, and (8) presentation of publishable (APA, etc.) results. This experience, conducted over the course of 8 weeks, provides a realistic and thorough simulation, and prepares graduate students to effectively create or modify their own instruments and produce high quality research products. The steps of the project and associated learning outcomes are represented in Table 1. In our application, students are required to submit three assignments that encompass the eight phases/steps in the survey research process.

**Table 1:** Project steps and associated learning outcomes.

**Simulation project steps**	**Learning outcomes and deliverables**
1. Project conceptualization	a) This first step involves determining the purpose of the survey, which latent constructs can and should be measured, and a brief literature review
2. Instrument design	a) Describe steps for conducting an effective pilot study, including use of cognitive interviews and focus groups for pilot testing
3. Presentation of a deployment plan, sampling considerations, and power analysis	a) Select and justify the most appropriate survey mode and develop procedures for survey administration b) Recognize the various sources that may contribute to total survey error and methods for reducing them (i.e., Tailored Design Method)
4. Creation of a codebook	a) Create codebooks for variables included in the survey b) Describe and present survey data organizational plan c) Describe the importance of quantitative data coding
5. Reliability/scale analyses	a) Recognize the different reasons for, and potential problems with, using various response scale options b) Evaluate scale reliability and gather validity evidence
6. Data reduction and scale exploration	a) Conduct a basic exploratory factor analysis b) Present inter-rater reliability assessment c) Evaluate response rate calculation, and demonstrate power analysis consideration
7. Writing research questions	a) Articulate appropriate research questions that can be addressed with existing date b) Link survey scales and collected data to overarching research problem
8. Data analyses	a) Demonstrate appropriate quantitative research methods to address a research question b) Determine if collected data fully supports the research questions outlined early in the project
9. Presentation and interpretation of results	a) Practice presentation of research results in APA style/format b) Gain facility with presenting and interpreting research results

## Survey simulation assignment procedures

The project begins with students conceptualizing a survey project idea. Readings, lessons, and class exercises are introduced to help students generate research ideas for capturing an unobservable behavioral, psychological, and perceptual construct (or series of related constructs). The assignment, and classroom instruction, relies on the seminal work presented by Dillman et al. (2014) and theoretical foundation of the Tailored Design Method and the Social Exchange Theory. Students are taught the value of utilizing validated instruments and scales but are not allowed to submit an existing validated instrument. The requirement to create a new instrument intentionally requires students to consider the importance of validating and testing instruments and sets the stage for provision of data and understanding how the design of an instrument impacts response rates and quality/completeness of data. As the project develops into later stages, students gain a first-hand perspective on the relationship between instrument design and data analyses.

The first deliverable (part 1) of the assignment includes a document representing the survey questions, a codebook spreadsheet, and a survey administration plan. Part two introduces students to data and reliability and the process of gathering validity evidence. Finally, part three requires students to conduct exploratory factor analysis (EFA) with their data, create new scale scores for their constructs, and answer a research question using their scale scores. Additionally, students are required to interpret their results and provide a reflection on the process that includes lessons-learned and considerations for future survey research. A full description of these components is offered in the next section along with a detailed guide for creating data for individual students.

### Project part 1: survey creation, administration plan, and codebook

To fully engage in the survey design and validation simulation, students are required to create a complete survey instrument including psychological/measurement scales, along with demographic information and key questions for data points pertinent to their study. The survey objective and research topic is determined by the student. Requiring students to generate a survey project related to their own research agenda creates a meaningful connection to the assignment and enhances their commitment to learning and depth of knowledge in topics important to them. Students are advised to generate at least five questions to measure two unobservable constructs of interest, and they must have a minimum of two scales on their survey. Existing scales can be used for the project, but not an entire instrument. An intentional aspect of the project is to require the first component to be due early in the term, before students are fully introduced to the importance of, and process for, validating and testing psychometric scales. An overarching learning outcome centers on the importance of instrument validation and the use of existing/validated scales over untested bespoke instruments. Novice researchers often assume that they can create an effective instrument and lack the knowledge that instrument development requires a proper validation and testing process (Dillman et al., 2014). This project is designed to dispel this assumption and demonstrate, through hands on experience, that hastily designed instruments are often ineffective.

Students are also required to provide detailed plans for administering their survey, including real-world budget and resource parameters. Students are asked to present context for their survey project, overarching research questions, and a plan for administering the survey. This component is accompanied by readings about sources of survey errors and survey administration considerations (e.g., Biemer and Lyberg, 2003; Dillman et al., 2014). The degree to which students thoroughly and successfully conceptualize and plan their survey administration (including incentives, sampling challenges) impacts how data is created and provided to students. This allows the instructor discretion related to the number of responses and level of missingness per item. For example, students who present unsophisticated plans and/or fail to account for sources of survey error can be provided higher levels of non-completion, missing data, and lower total responses. At this stage of the project, it is recommended that students have clear parameters regarding the number and type of items on the instrument. Creating data for students is time intensive, therefore we advise students to present no more than 25 total items, including a slate of demographic items and critical information related to the research questions (e.g., employment information, affinity group, etc.).

Along with the survey instrument, students are required to submit a codebook for their items. Codebooks are used to document the values associated with the answer options for a given survey question (Carley-Baxter, 2008). This process imparts upon student the importance of providing a guide for coding responses and serves as documentation of the layout and code definitions of the eventual data file. Students are required to provide specific details about the data including the variable names, whether the variable is numeric or character (string), and the format of numeric variables. Additionally, the question text and answer categories should be clearly documented along with frequencies of each response option. In class students are provided example codebooks from both small- and large-scale survey research projects along with a short in-class exercise the demonstrates the importance of codebooks. This component of the assignment submission is important for students to understand broadly but is also necessary for creating data for each student's project.

#### Data creation

After receiving the first part of the assignment, instructors must now create datasets for students to use in completing parts two and three of the project. To create data we use the online mock data generator, Mockaroo (https://mockaroo.com/). Mockaroo is a free online data generator that is designed to provide realistic data based on user specifications. Mockaroo does not require programming. Instead, it relies on a user-friendly interface for entering specific field parameters, definitions, and types. The free version of the application currently provides up to 1,000 rows of data for each dataset generated. Benefits of using the program include the ability to control responses, control/introduce error conditions, design your own mock APIs, and create associations among the data.

Although the program has a host of templated item types (i.e., animal names, car models, and cryptocurrencies, etc.), the “numeric”, or “custom list” item types have the most application to social science research where perceptions, frequencies, and rating scales are collected. The “custom” option allows for inputting values for the program to choose from randomly (e.g., Likert scale 1, 2, 3, 4, 5). The application also allows the user to base responses around a user specified distribution. For example, a five-point Likert item can be generated with a mean of 3.25 across all responses on a given item. This allows the user/instructor to create mean differences on an item (or multiple items) based on demographics (i.e., race, gender, age, etc.). Additionally, the user can create correlations between items by using the “Custom Distribution” function and creating “rules” while creating individual item responses. Carefully creating data with Mockaroo allows a user/instructor to create similar (or dissimilar) item distributions within a block of questions measuring a given construct. This is particularly important when presented with reverse coded items, or scales that represent convergent or divergent validity. Finally, the user can determine the percentage of missing data when creating each item. Missing data is common across social science research and levels of missingness can be applied based on item characteristics (e.g., later questions on long surveys, confusing questions, privacy concerns, etc.). The ability to specify distributions within and across items/scales provides for a much more realistic and nuanced dataset for students to utilize for the remaining project components. Creating associations and patterns within the data also provides a base for realistic data analyses and outputs that mimic real world conditions. The following is an outline of the process for creating data within Mockaroo:

*Step one: create account*

Create free account with Mockaroo, which allows for 1,000 rows of data and hundreds of data fields (variables). Once a profile has been created the user can create and save “schemas” that can be modified and accessed later.

*Step two: import data field headers*

Users can import field/variable names to save time and create the foundation for a schema that can be edited. The field headers have data restrictions and use of uppercase, spaces, and special characters will create errors. In the codebook assignment guide, students are required to submit variable names according to data type parameters in Mockaroo.

*Step three: define variable parameters (type and options)*

Users can explore a range of data types when creating each variable. Mockaroo allows for multiple data types and pre-defined categories. Most categories are not useful for social science research. The most useful field type is “custom”, which allows the user to enter specific categorical responses, or create a categorical numeric scale (e.g., a five-point response Likert-type scale). To skew a higher rating for a survey item, a user can enter “1, 2, 3, 4, 4, 4, 5, 5” as the response options, providing a higher proportion of responses in the upper range of the response scale. To associate items, users must choose “custom” as the item type. Alternatively, if a continuous/numeric scale is appropriate, users can choose “Normal Distribution”, or “Numeric” and specify mean and standard deviation for the item. Specific decimal points or whole numbers can also be specified.

*Step four: create data relationships*

To develop relationships among the items on the instrument, copy paste the response distribution for the group of items that should relate to one another. Another option is to utilize the distribution matrix tool, accessed by clicking on the “Create a Custom Distribution” button identified as a bar-chart icon with the item options tool. Creating structure and patterns when creating the items allows for a logical result when investigating for patterns within the data, e.g., exploratory factor analysis, and eventual correlational analyses.

*Step five: finalize survey and create data*

Once all the variables, and options are set, save the schema and specify the number of data rows that will be generated. Additional options include various file formats outputs (e.g., csv, excel, SQL, XML), and whether to include the header row. Finally through clicking “Preview” and “Download Data”, users can inspect the data prior to sharing this back to the students.

### Project part 2: descriptive analyses, validity, and reliability

Along with receiving feedback and grading of part one of the assignment, students receive their data file. For the second part of the survey project students are required to review their data and conduct key descriptive statistics, missingness evaluation, examine for outliers, and consider any coding/recoding data cleaning steps. Additionally, at this stage in the course, students are introduced to data reduction techniques and scale reliability (readings and lab exercises in class). As reinforcement of these lessons, students are required to conduct an EFA with their survey data. This component introduces students to factor extraction methods, data rotations, interpretation of factors (i.e., scree plots, eigenvalues), and fundamentals of data reduction. Conducting an EFA allows for understanding of the underlying structure of the survey items and how they relate to each other, thus researchers can build confidence that the survey measures the constructs of interest (Boateng et al., 2018). Additionally, the EFA process helps in assessing the convergent validity (the degree to which items that are supposed to measure the same construct are correlated) and discriminant validity (the degree to which items that are supposed to measure different constructs are not correlated) of the survey items (Shultz et al., 2021). Finally, through examining factor structure, students in the course can identifying redundant or irrelevant items that do not contribute to measuring the intended constructs. Removing such items improves the clarity and precision of the survey instrument, thereby enhancing its validity (Shultz et al., 2021).

Once students sufficiently evaluate factor scores and decide on item groupings, they then conduct scale reliability analyses for groups of items and create scale scores where appropriate. Through analyzing reliability measures and considering data reduction, students improve their survey design skills and build confidence for future survey research. Students are made aware that data has been generated and that items may not associate the way they have hypothesized, and that this may also be the case using real data. This stage of the simulation process is paired with readings on scale validation, reliability, and the complex nature of social/behavioral science data. For example, students read Harpe (2015) and Boateng et al. (2018), for depth and sophistication involved in scales development, validation, and working with Likert-type items.

### Project part 3: data analysis, interpretation, and survey reflections

For the final component, students are required to apply intermediate or higher quantitative techniques to answer two quantitative research questions using their data. Students must present a results section write-up using APA as a style guide, but are not required to write a full literature review and discussion linked to their project. Given the methodological focus of the course, readings and assignments cover measurement and assessment topics and focus on building skills with quantitative analyses.

Additionally, and importantly, students are required to reflect on the process and identify specific areas for improvement and continued focus/development related to survey research. In the reflective component, students are asked to present a plan and considerations to improve question wording, formatting, order of questions, consider response processes, and improve the survey overall based on what they have learned from the simulation. Students are also required to revisit the Dillman et al. (2014) text, and reconsider potential sources of survey error and what changes they might make if administering the survey in a real-world application. At this final stage, students are also reminded of the iterative nature of the validation process, and the need for periodic updates based on emerging research or changes in the application context. Although Confirmatory Factor Analysis is not required as part of the assignment, course readings introduce this next step as a validation measure.

## Limitations and challenges

The simulation is not a substitute for pilot testing a new instrument with real survey respondents. Rather, this exercise is analogous to the drafting process of any creative work. The benefits include far less resources and reduction/elimination of engagements with research participants. However, genuine challenges in the process exist. Firstly, the time commitment to creating data for students is significant. Our experiences suggest that creating data for an individual student can range from 1 to 2 h. Applying this simulation with large enrollment courses is impractical, thus limiting this exercise to small enrollment graduate seminars, or a course with a graduate teaching assistant who could assist the instructor (e.g., TA could create preliminary data schemas for the instructor to check over and create the final dataset). Another efficiency option might be introduction of a lab exercise that requires students to create their own data schema (or create data for each other) that could be reviewed and finalized by the instructor.

Next, challenges relate to the nature of simulated data and difficulty in presenting true-to-life data associations. Mockaroo has tools built to create specific distributions, but mistakes can easily occur when creating data for multiple students who may not always communicate effectively through their codebooks, survey plans, etc. Lastly, there is risk in introducing this tool to students who may decide later to create a falsified dataset for other research projects, e.g., theses, dissertations, etc. The ability to create a realistic dataset might be a temptation for students who find difficulty at the dissertation phase of study, thus a focus on ethical practices in research in this assignment is encouraged.

Finally, the impact of this work might be more clearly presented with a formal assessment of student performance and understanding of concepts before and after the simulation project. However, given that no initial IRB approval was sought as part of the course administration, pre- and post-test data was not collected beyond course outcomes via traditional grading of assignments.

## Discussion

Although our application focuses on survey design, using simulated data has numerous applications for developing researchers and new research techniques. For more advanced programmers, R Studio is free to use, and open-source sample code can be adopted to create simulated data. For a less robust experience with survey simulations, Qualtrics (a survey data collection service) provides a function for creating “test data” for survey questions. This tool is easy to apply once a survey is built in Qualtrics, however the responses are truly random and will not be useful for creating a realistic data analysis exercise. Finally, other statistical packages (e.g., SAS), provide guides and coding language for creating simulated data, but these programs are proprietary and expensive to purchase.

### Determining if simulated data is appropriate

Using simulated survey data, researchers have control over the characteristics of the data, enabling them to conduct controlled experiments and manipulate variables to test specific hypotheses or scenarios. Simulated data can mimic various population characteristics, response patterns, and survey designs, providing flexibility in experimental design. Additionally, privacy concerns associated with using real participant data are eliminated, as researchers can freely manipulate and share simulated datasets without compromising confidentiality. Simulated data are also generally more cost-effective than collecting real survey data. And, as demonstrated in this paper, simulated data can be valuable in offering students and novice researchers practical experience in survey design, data analysis, and interpretation.

However, there are also drawbacks to using simulated survey data. Simulated data will not fully capture the complexity and variability of real-world survey responses and should never be used to generalize findings to real populations or settings. Additionally, simulated data rely on underlying assumptions and models that may not accurately reflect the true data-generating process, affecting the reliability of simulated results. Generating high-quality simulated data requires time, expertise, and effort to develop realistic models and simulate plausible response patterns, particularly for complex survey designs and population characteristics.

Using existing data collected from real surveys has clear advantages. Existing data reflect actual responses from sampled populations, providing a realistic representation of survey outcomes and population characteristics. Moreover, using existing data can be more cost-effective (often free) and time-efficient than simulating or collecting new survey data, especially within the limited setting of an academic term. Additionally, findings based on existing data necessarily have greater external validity. Use of existing data should be considered if course learning outcomes emphasize conducting analyses and interpretations over survey/instrument design objectives.

In creating a instrumentation curriculum, instructors should also consider possible constraints related to the use of existing datasets. Design characteristics and privacy/confidentiality concerns may limit students' ability to manipulate variables or test specific hypotheses compared to simulated data. Access to existing datasets may also be restricted or limited by data providers, requiring researchers to navigate data access protocols or negotiate data-sharing agreements.

## Conclusion

Working with existing survey data to learn quantitative techniques ignores the survey creation and validation process and the complexity of the full survey research experience (Fellers and Kuiper, 2020). Furthermore, having students create a survey on a topic that is of interest to them helps connect them to the research topic and creates a buy-in, which has been linked to doctoral student retention (Bair and Haworth, 2004; Litalien et al., 2015; Hanson et al., 2022). This process allows students to understand the consequences of creating hastily designed survey and provides students the full experience of working with realistic and messy data (Witte, 2017).

This project is designed to introduce students to survey research through engagement in a holistic project (design instrument, present a deployment plan, codebook, reliability/scale analysis, factor analysis, question analysis/stats, write up). Through simulating the use of a new instrument, researchers-in-training can reduce efforts and costs associated with multiple rounds of pilot testing, tuning of scales, and reduce survey design/implementation errors. Through simulation, research questions can be asked and fully answered, giving novice researchers a full research experience. Anecdotally, students at our institution gained confidence and successfully piloted and validated new instruments after completing this project.

## Data availability statement

The original contributions presented in the study are included in the article/supplementary material, further inquiries can be directed to the corresponding author.

## Author contributions

PM: Conceptualization, Data curation, Methodology, Writing – original draft, Writing – review & editing. ET: Writing – original draft, Writing – review & editing.
